# Hepatitis E virus infection in pregnant women, Argentina

**DOI:** 10.1186/s12879-020-05087-3

**Published:** 2020-05-24

**Authors:** Gabriela Tissera, María Cecilia Lardizabal, Sofía Belén Torres, Anabella Clara Fantilli, Maribel G. Martínez Wassaf, Fernando Venezuela, Raúl Capra, Domingo C. Balderramo, Claudia Travella, Viviana E. Ré, María Belén Pisano

**Affiliations:** 1 Instituto de Virología “Dr. J. M. Vanella”, Facultad de Ciencias Médicas, Universidad Nacional de Córdoba (UNC), Enfermera Gordillo Gómez, s/n (without number), X5016 Córdoba, Argentina; 2 Servicio de ginecología y obstetricia, Hospital Privado Universitario de Córdoba, X5016 Córdoba, Argentina; 3 Consejo Nacional de Investigaciones Científicas y Técnicas (CONICET), C1425FQB Buenos Aires, Argentina; 4 Laboratorio de Análisis Clínicos Especializados (LACE), X5016 Córdoba, Argentina; 5 Laboratorio de análisis clínicos, Hospital Privado Universitario de Córdoba, X5016 Córdoba, Argentina; 6 Servicio de gastroenterología y endoscopía digestiva, Hospital Privado Universitario de Córdoba, X5016 Córdoba, Argentina; 7 Instituto Universitario de Ciencias Biomédicas de Córdoba (IUCBC), X5016 Córdoba, Argentina

**Keywords:** ARGENTINA, HEPATITIS E VIRUS, PREGNANT WOMEN, PREVALENCE

## Abstract

**Background:**

Hepatitis E virus (HEV) infection is an important cause of acute hepatitis worldwide. In pregnant women, HEV can cause more severe symptoms, with high rates of fatal hepatic failure in endemic countries. However, HEV prevalence and circulation among pregnant women from South America is almost unknown. We aimed to investigate HEV infection in pregnant women for the first time in Argentina.

**Methods:**

IgG and IgM anti-HEV antibodies and RNA-HEV were investigated (by ELISA assays and RT-Nested-PCR, respectively) in 202 serum samples from pregnant women collected in the central region of Argentina between 2015 and 2017. A control group of 155 non-pregnant women was included (year 2018).

**Results:**

The IgG anti-HEV positivity rate was 8.4% (17/202), higher than the 2.6% (4/155) obtained for the non-pregnant women control group, and showing association between pregnancy and HEV infection (*p* = 0.023, OR = 3.5, CI95% = 1.1–10.5). Women younger than 25 years old presented higher levels of antibodies, and there were no differences in the prevalences between trimesters of pregnancy. Two samples were reactive for IgM anti-HEV, showing recent infections, although no symptoms were registered in these patients. All samples were negative for RNA-HEV amplification.

**Conclusions:**

HEV produces infections in pregnant women from Argentina, alerting health teams to consider it as a possible cause of liver disease.

## Background

The hepatitis E virus (HEV) (specie *Orthohepevirus A*, genus *Ortohepevirus*, family *Hepeviridae*) is an important pathogen of worldwide distribution, causing acute hepatitis, transmitted mainly by the fecal-oral route [1, 2]. HEV is a non-enveloped virus with a positive sense single stranded RNA genome. According to its genetic variability, it has been classified into 8 genotypes [1–8], from which 5 of them can infect humans (1 to 4 and 7) [1, 3].

Clinical presentations of HEV infection are varied: immunocompetent individuals can develop asymptomatic or mild infections, while immunocompromised patients, pregnant women, transplant patients or those with underlying liver problems can lead more severe illnesses, including fulminant hepatic failure (FHF) and chronic disease [4]. Particularly in pregnant women, severe acute liver disease and progression to FHF have been registered when the infecting genotype is 1, which may result in fetal and/or maternal mortality, abortion or premature delivery [5]. Because of this, pregnant women have attracted more attention regarding HEV infection, mainly in endemic regions, where seropositivity rates in this group of patients reach values as high as 68% [4].

In South America, there are very few studies about HEV detection in pregnant women, performed in Venezuela and Brazil in the 1990’s decade, which report IgG anti-HEV seroprevalences of 1.0, 1.3 and 1.9% [6, 7]. After several years, a study from Brazil reported a much higher prevalence of 19% in the pregnant women group, showing no significant differences with blood donors from the same region [8].

In Argentina, acute hepatitis E cases are reported sporadically. HEV-RNA genotype 3 (HEV-3) detections have been performed in pigs and environmental samples, and two chronic cases were documented [9]. Strikingly, an acute hepatitis E case in a 41 years old woman, who had had a spontaneous abortion a month before onset of symptoms, was registered in Mendoza province, at the West region of the country [Bussetti et al. 2017, personal communication]. Despite this background, there are no epidemiological studies about HEV in pregnant women in Argentina. Having data about the circulation of HEV in this population would contribute to the knowledge and to provide appropriate public health recommendations in our region. For this reason, we aimed to investigate the local circulation of HEV in this group of patients.

## Methods

This is an anonymous, descriptive, retrospective, non-interventionist study in which 202 serum samples from pregnant women (median age: 30 years; range: 18–43 years) collected and stored in 2 health centres from Córdoba (central region of Argentina) between 2015 and 2017, were analysed for IgG and IgM anti-HEV detection by ELISA assays (Diapro, Italy; diagnostic specificity ≥98%, diagnostic sensitivity ≥98%). Positive samples were tested for HEV-RNA amplification by RT-Nested PCR, amplifying a 348 bp fragment of the ORF-2 region [10]. Only the following data were available: date of sampling, age, neighbourhood and trimester of pregnancy.

Additionally, 155 serum samples from non-pregnant women who attended health care centres from Córdoba city for a routine control during April and May 2018 were retrospectively analysed as a control group.

Prevalences were expressed as percentages. To assess the association between individual variables and IgG anti-HEV, we used independent t or chi-square tests. Exact 95% confidence intervals (CIs) were utilized. Statistical significance was defined at *p* < 0.05. The statistical package Stata 13.0 was used.

This study was approved by the Training and teaching Committee of the Hospital Rawson and the Ethics Committee of the Hospital Privado Universitario de Córdoba (protocol HP 4–281), in accordance to the specifications of the Ministry of Health of the Province of Córdoba, Argentina (Law N°9694). Since this was a retrospective study, informed consent was not required.

## Results

**Fig. 1:**
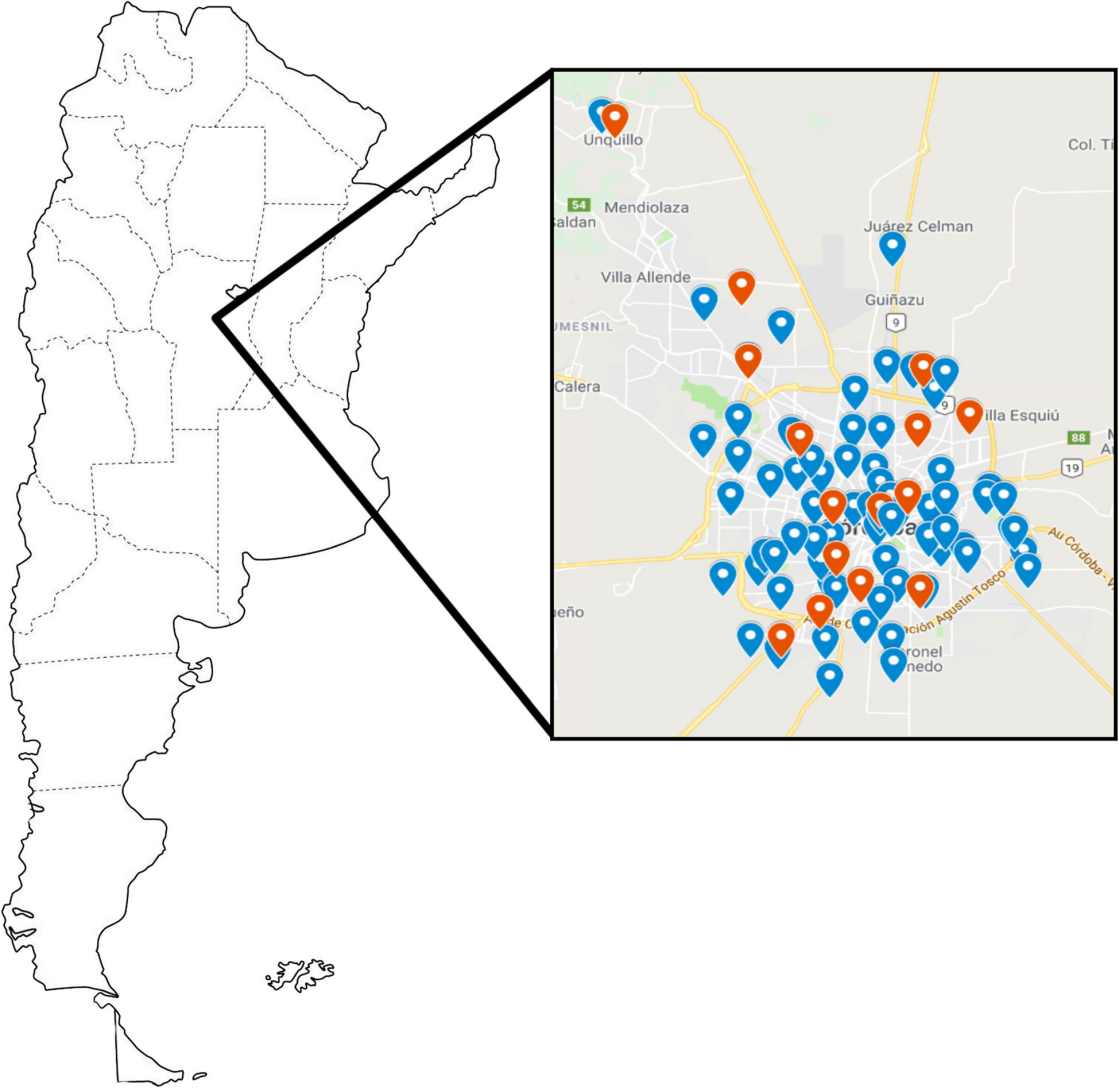
Map showing the geographical location of the samples analyzed during this study in Córdoba province, Argentina. The blue bullets indicate the location of the negative anti-HEV IgG samples, while the red bullets indicate the location of the positive anti-HEV IgG samples. The image of the map was taken from Pinterest, under written consent from the author

A total of 202 serum samples from pregnant women were analysed for HEV. The global IgG anti-HEV prevalence obtained in this population was 8.4% (17/202), higher than the 2.6% obtained in the control group (4/155) (*p* = 0.023). Statistical association between pregnancy and HEV infection was found (OR = 3.5, CI95%: 1.1–10.5). Distribution of the positive samples among and around the city was homogeneous, without a distribution pattern (Fig. 1), and no significant difference was observed in the HEV seroprevalence according to the neighbourhood. Although the prevalence was higher in the second trimester (14%), this difference was not statistically significant (*p* = 0.42) (Table 1). When dividing the samples into group ages, surprisingly, the ≤25 years old group showed the higher seropositivity value (16.7%, *p* = 0.032) (Table 1), showing statistical association between low age of pregnancy (≤25 years old) and HEV seroprevalence (OR = 3.2, CI95%: 1.2–8.9). No significant difference was found in the HEV seroprevalence when analysing the samples according to the studied years.

**Table 1:** Number of samples analysed, IgG-anti HEV positive samples and seropositivity rates obtained in pregnant women from Argentina according to the variables analysed

Variable		N° samples analysed	N° IgG anti-HEV positive samples	HEV seropositivity (%)	*P*-value
Age (in years)	≤25	48	8	16.7%	*p* = 0.032*
	26–35	112	6	5.4%	
	≥36	42	2	4.8%	
Trimester of pregnancy^a^	1st	75	6	8.0%	*p* = 0.421
	2nd	57	8	14.0%	
	3rd	65	3	4.6%	

^a^This data was not available in 5 samples

*Statistically significant difference

From the 17 positive samples for IgG anti-HEV, 2 were reactive for IgM anti-HEV, evidencing acute (recent) infections. One of them, belonged to a 36 years old patient in the second trimester of pregnancy, with pork and fish consumption habits less than once a week, without history of transfusions (in this case epidemiological data could be obtained); while the other IgM positive sample belonged to a 18 years old patient in the first trimester of pregnancy, with no other available data. These samples resulted negative for HEV-RNA amplification. None of these patients had a history of symptomatic acute hepatitis, so mild or sub-clinical infections would have occurred.

## Discussion

HEV is known to produce acute hepatitis worldwide, with some complications in co-morbidity patients. In pregnant women, fatality rates are increased in endemic regions, being as high as 20–30% [11]. This may be related to several possible factors, such as differences in immune and humoral factors occurring during pregnancy, the genetic and environmental factors with its occurrence in certain developing countries, and the infecting HEV genotype [12].

In South America the overall epidemiology of HEV has been little studied, and the burden of the disease remains largely unknown [9]. There are very few studies about HEV detection in pregnant women, most of them performed in the 1990’s decade (IgG anti-HEV seroprevalences of 1.0, 1.3 and 1.9%) [6, 7]. Our results show, for the first time, a HEV seropositivity rate of 8.4% in pregnant women from Argentina, similar to the prevalences found in developed countries for the same population, such as France (7.7%) [13], but showing a great difference with a recent Brazilian seroprevalence value of 19% [8]. This could be due to many reasons: differences in the HEV local circulations, the level of public health and hygiene of each region [4], and the kit utilized for the HEV antibody detection, since Wantai kit (used in the Brazilian study) has been reported to be more sensitive [9, 14], yielding higher seroprevalence values. We found a higher HEV prevalence in pregnant women than the control group (non-pregnant women, 2.6%), as well as than general population in the same region, which has been previously reported to be 4% [10]. This could be a consequence of the decrease in some of the immune responses during pregnancy [15], together with hormonal changes [12] (increase of steroid hormones), which facilitates a scenario prone to infections (and higher levels of antibodies). It would be important to obtain data of HEV circulation in pregnant women from other regions of our country, since there could be variations in seropositivity rates among different geographical regions, which reflect differences in the status of public health and hygiene, risk factors, and routes of transmission, as reported in other countries [16].

When dividing the samples into group ages, surprisingly, the group ≤25 years old had the highest seropositivity rate. This is contrary to what we expected and other studies have reported: that seroprevalence increases with age, probably due to more exposure to the virus [4, 5, 10]. One explanation for our results could be that women belonging to the ≤25 years old group came from low income neighbourhoods, which could indicate poor sanitary conditions and an increased viral transmission. It would be necessary to analyse a higher number of samples belonging to different neighbourhoods (with different income levels) to achieve a conclusion regarding an association between the age of the patients and the HEV serostatus.

We demonstrated the presence of acute infections during pregnancy in Argentina (individuals with positive IgM anti-HEV), showing viral circulation among this group of patients. The absence of risk factors in one of the positive samples (such as consumption of pork or fish, contact with pigs, transfusions) would reveal alternative routes of transmission, such as contact with water contaminated with the virus, taking into account that HEV has been detected in local recreational aqueous matrices [17].

Unfortunately, RNA-HEV could not be amplified in any sample, so the infecting genotype could not be determined. It is known that HEV genotype 3 (HEV-3) circulates in Argentina [9], so it is possible that this genotype would be the responsible for infections in the pregnant women studied. This could be a reason for the occurrence of asymptomatic infections, since severe hepatitis and FHF cases have been reported as a consequence of HEV genotype 1 (HEV-1) infections in other parts of the world [12]. However, it is important to continue monitoring acute liver diseases in this population due to the detections of HEV-1 reported in the bordering country of Uruguay [18, 19]. This situation would lead to the entrance of HEV-1 to Argentina (from Uruguay), or to the HEV-1 infection of Argentine pregnant women who visit Uruguay (the Uruguayan coasts are a holiday destination for many Argentine people).

## Conclusions

Our results show that HEV produces infections in pregnant women in Argentina, alerting physicians as a possible cause of hepatitis in this population. A close follow-up in pregnant women should be necessary to prevent HEV infections, as well as to early detect severe infections in our country.

## Data Availability

The datasets used and/or analysed during the current study are available from the corresponding author on reasonable request.

## Abbreviations

HEV: Hepatitis E virus
RNA: Ribonucleic acid
RT-Nested PCR: Retro-transcription nested polymerase chain reaction
Ig: Immunoglobulin
FHF: Fatal hepatic failure
ELISA: Enzyme-linked Immuno-sorbent assay
